# A Soluble Expression Construct of the Isolated Catalytic Domain of *Plasmodium falciparum* ATP4 Exhibits ATPase Activity Independent of a γ‐Phosphate Receiving Aspartate

**DOI:** 10.1111/mmi.15358

**Published:** 2025-03-17

**Authors:** Timo Beyer, Jesko Caliebe, Lara Kähler, Eric Beitz

**Affiliations:** ^1^ Department of Pharmaceutical and Medicinal Chemistry Christian‐Albrechts‐University of Kiel Kiel Germany

**Keywords:** ATPase, malaria, proton, sodium, structure–function relationships

## Abstract

The sodium/proton‐exchanging ATPase of *Plasmodium falciparum* malaria parasites, PfATP4, is an emerging drug target. Inhibition results in detrimental cell swelling due to cytosolic accumulation of sodium and alkalization. PfATP4 is a sodium‐releasing type II P‐type ATPase restricted to apicomplexan parasites. Experimental data on structure–function relationships of the isolated protein are absent. Here, we produced and purified the soluble catalytic domain of PfATP4 and evaluated kinetic properties by in vitro phosphate colorimetry. The protein exhibited Mg^2+^‐dependent ATPase activity at the same order of magnitude as the native cellular PfATP4 and was insensitive to the presence of sodium. AlphaFold 3‐based structure and ATP/Mg^2+^ interaction predictions identified key residues of the nucleotide binding domain (Lys619, Lys652, Arg703). Replacement of the lysines by methionine decreased the enzymatic activity to one quarter. Individual mutation of the putative Mg^2+^‐coordinating Asp865 of the phosphorylation domain was tolerated, while a joint replacement with Asp869 decreased ATPase again to one quarter. Mutation of the putative γ‐phosphate receiving Asp451 maintained the rate of P_i_ release. Our data attribute typical functional roles for P‐type ATPases to the basic and acidic residues of the soluble PfATP4 catalytic domain and show that its ATP hydrolysis is independent of phosphorylation of Asp451.

## Graphical abstract

Biochemical data on the isolated Na^+^/H^+^‐exchanging ATPase, PfATP4, from the malaria parasite are missing. Here, we expressed and purified the nucleotide and phosphate binding domains as a soluble protein and identified key residues by enzymatic characterization of generated point mutants.

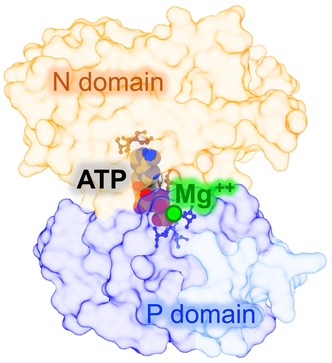

**Abbreviation**
PfATP4: *Plasmodium falciparum* sodium/proton exchanging ATPase

## 1 Introduction

**FIGURE 1:**
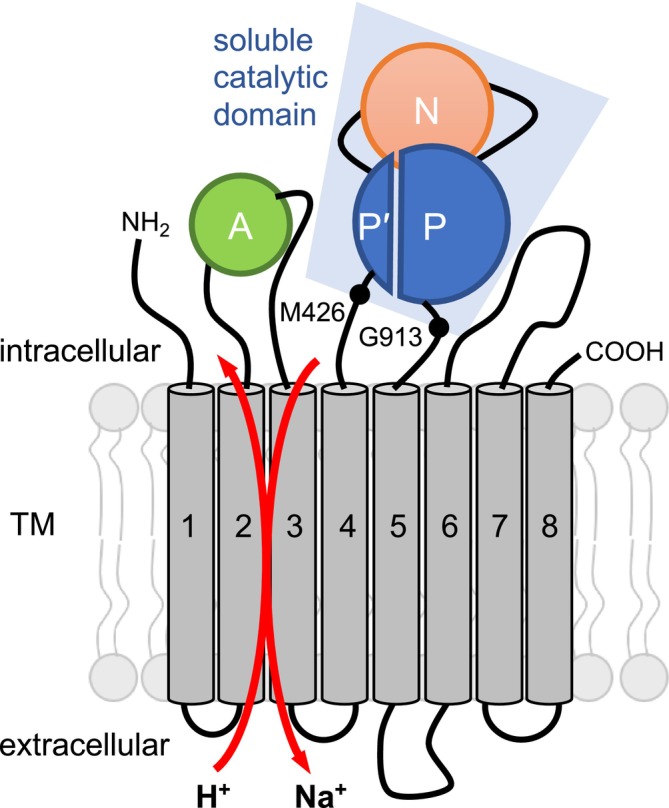
Schematic model of the sodium/proton exchanging PfATP4 and soluble expression construct comprising Met426 to Gly913. PfATP4 is predicted to have eight transmembrane spans and a cytosolic actuator (A) domain, a nucleotide‐binding (N) domain, and a split phosphorylation (P′/P) domain.

Sodium homeostasis is a crucial prerequisite for the survival of *Plasmodium falciparum* malaria parasites. During host infestation, the parasite resides within human erythrocytes, encompassed by a parasitophorous vacuole that is highly permeable to low molecular weight solutes and ions (Matz et al. 2020; Spielmann et al. 2012). Parasitization further leads to the establishment of new permeability pathways in the erythrocyte plasma membrane, enabling influx of Na^+^ and efflux of K^+^ along their respective gradients, resulting in cytosolic levels similar to those in the extraerythrocytic plasma (Ginsburg et al. 1985; Kirk et al. 1994; Staines et al. 2001). The parasite is therefore directly exposed to a steep inward gradient of Na^+^. In order to maintain a low cytosolic Na^+^ level, plasmodia rely on the continuous action of a primary efflux pump, PfATP4. PfATP4 mediates the ATP‐driven extrusion of Na^+^ in exchange with protons, thus preventing a detrimental alkalinization of the cytosol as well as cell swelling and premature schizogony, which would eventually cause the death of the parasite (Spillman et al. 2013; Dennis et al. 2018; Das et al. 2016). PfATP4 has been annotated as a P‐type ATPase belonging to a novel ATP4‐type subfamily restricted to apicomplexan parasites, which is most closely related to type IID (Lehane et al. 2019). Nonetheless, all P‐type ATPases share a common mechanism of transport. The catalytic complex consists of three domains: actuator‐, nucleotide‐binding, and phosphorylation domain (Palmgren and Nissen 2011; Figure 1). During the catalytic cycle, ATP binds to the nucleotide‐binding domain and reversibly phosphorylates a conserved aspartate residue within the phosphorylation domain (Figure 1). The subsequent dephosphorylation is catalyzed by the actuator domain, coupling the catalytic domain to the transmembrane region for sodium/proton transmembrane transport (Dyla et al. 2020).

PfATP4 is predicted to span the parasite plasma membrane eight times (Figure 1; Spillman and Kirk 2015). In analogy to other P‐type ATPases, the cytosolic domain carries conserved regions that likely form a nucleotide binding (N) domain, a phosphorylation (P′/P) domain, and an actuator (A) domain (Figure 1). Due to its essential role in parasite survival and the absence of functional homologues in mammalian hosts, PfATP4 has been under scrutiny as a druggable antimalarial target (Meier et al. 2018; Dick et al. 2020).

To date, several inhibitor classes related to PfATP4 have been identified, with the spiroindolone cipargamin as the most promising candidate that has successfully passed phase II clinical trials (Zagórska and Jaromin 2023; Schmitt et al. 2022; Spillman and Kirk 2015). However, functional studies are hitherto restricted to cell‐based assays conducted with *P. falciparum* parasites or *Xenopus laevis* oocytes, and the elucidation of the catalytic cycle and protein structure has been hampered due to the lack of isolated protein.

In this study, we expressed a cytosolic section of PfATP4, comprising the N and P′/P domains as a functional soluble protein and assessed its kinetic properties. We established structure–function relationships through alteration of conserved residues within the catalytic center.

## 2 Results

### 2.1 The Soluble Protein Comprising the N and P Domains of PfATP4 Is Functional

**FIGURE 2:**
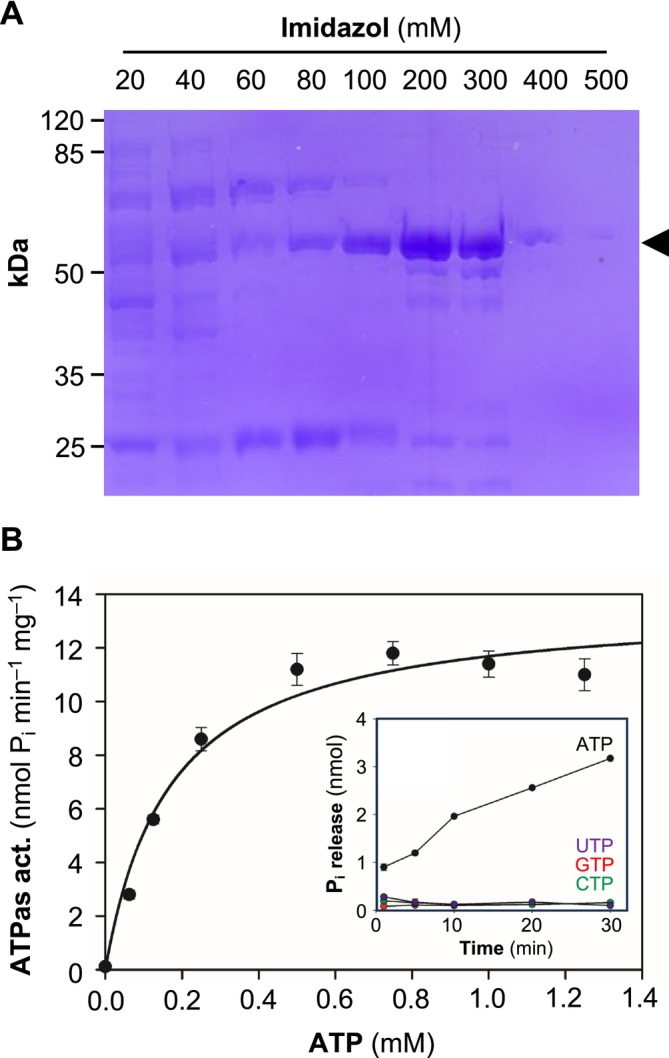
Purification and initial functional analysis of the soluble catalytic domain of PfATP4. (A) Ni^2+^‐affinity chromatography of PfATP4 Met426‐Gly913 with the C‐terminal His_6_‐tag. The arrow head indicates the protein of interest at the expected size of 54 kDa. (B) ATP‐dependent activity of the purified wildtype domain. Data represent mean ± SEM (*n* = 3), and were fitted using the Michaelis–Menten equation. The inset shows the nucleotide selectivity as the release of P_i_ over time at a 1 mM concentration of the respective nucleotide (mean ± span, *n* = 2).

Initially, we expressed the cytosolic domain of wild‐type PfATP4, comprising the cytosolic loop from Met426 to Gly913 (UniProt #A0A143ZZK9; Figure 1) in *E. coli* . Subsequent affinity purification via a C‐terminal His_10_ tag yielded up to 2 mg of protein from 500 mL of culture (Figure 2A). The protein exhibited the expected size of 54 kDa and eluted mainly in the 200–300 mM imidazole fractions, which were pooled and concentrated.

In order to evaluate the enzymatic activity of the cytosolic domain and to determine kinetic properties, ATP hydrolysis from 10 μg purified protein was measured as the release of P_i_ over 30 min at a Na^+^ concentration of 150 mM and a pH of 7.2 (Figure 2B). The ATPase activity increased with increasing ATP concentrations, reaching a plateau at 0.75 mM ATP. Fitting to the Michaelis–Menten equation yielded a K_m_ of 0.17 mM and a v_max_ of 13.6 nmol P_i_ min^−1^ mg^−1^ protein (Table 1). To exclude protein‐independent hydrolysis artefacts by the assay, we measured P_i_ release in the presence of PfATP4 from 1 mM ATP, GTP, CTP, and UTP (Figure 2B, inset). Only ATP as a substrate of PfATP4 showed an increasing release of P_i_ over time, ensuring enzyme‐catalyzed hydrolysis rather than chemical instability of the substrates.

**TABLE 1:** Substrate affinity (K_m_) and hydrolysis rate (v_max_) of the wild‐type and mutant PfATP4 catalytic domain (mean ± SEM for *n* ≥ 3, and mean ± span for *n* = 2).

PfATP4	K_m_	V_max_
Met426‐Gly913	(mM)	(nmol P_i_ min^−1^ mg^−1^)
Wildtype (150 mM Na^+^; *n* = 3)	0.17 ± 0.04	13.6 ± 0.9
Wildtype (0 mM Na^+^; *n* = 3)	0.10 ± 0.02	15.4 ± 0.7
K619M (*n* = 2)	0.23 ± 0.03	4.2 ± 0.2
K652M (*n* = 3)	0.15 ± 0.06	3.3 ± 0.3
R703M (*n* = 2)	0.23 ± 0.02	11.1 ± 0.2
K619,652 M (*n* = 3)	0.41 ± 0.23	3.5 ± 0.8
D865N (*n* = 3)	0.18 ± 0.04	12.3 ± 0.8
D865,869 N (*n* = 2)	0.33 ± 0.18	3.2 ± 0.6
D451N (*n* = 2)	0.18 ± 0.05	11.6 ± 0.8
D451N/D865,869 N (*n* = 4)	0.14 ± 0.06	3.9 ± 0.5

### 2.2 Enzymatic Characterization of the PfATP4 N and P Domains

**FIGURE 3:**
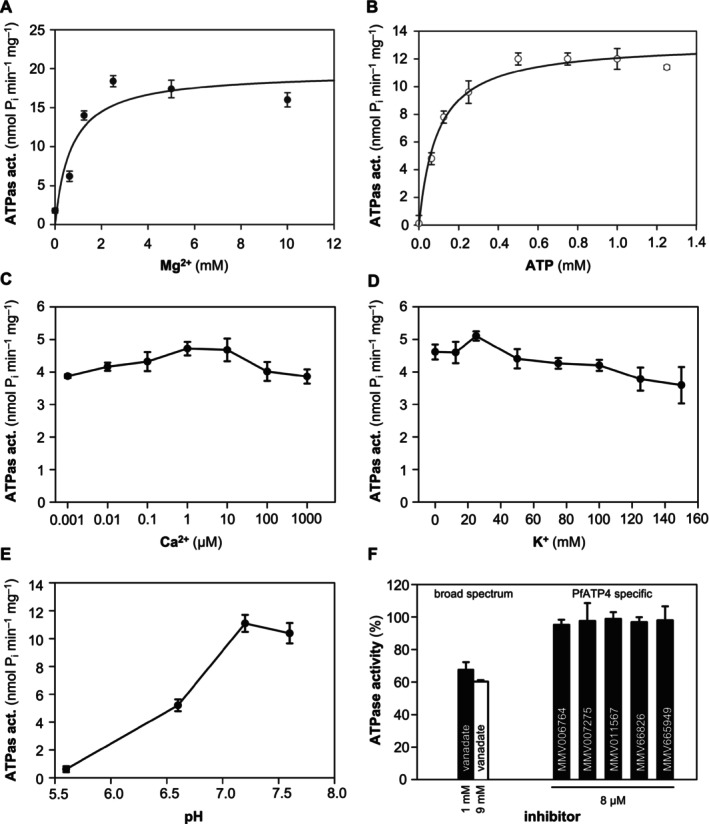
Ion and pH dependence of the PfATP4 Met426‐Gly913 ATPase activity plus inhibition. (A) Shown is the effect of Mg^2+^ variation at a constant ATP of 1 mM. (B) ATPase activity in the absence of Na^+^, and at various Ca^2+^ (C) and K^+^ concentrations (D) or pH (E) at 1 mM ATP. (F) Effect of inhibitors on PfATP4 activity Data are shown as mean ± SEM (*n* = 3 or *n* = 2 for inhibitors) fitted with the Michaelis–Menten equation.

Since Mg^2+^ dependence is a key feature of P‐type ATPases, we determined the ATPase activity at different Mg^2+^ concentrations (Figure 3A). ATP hydrolysis strongly increased with rising Mg^2+^ concentrations, reaching a maximum at 2.5 mM Mg^2+^. Since the native full‐length PfATP4 required Na^+^ for ATPase activity, we also tested the ATPase activity in the absence of Na^+^ (Figure 3B). With a K_m_ of 0.10 mM and a v_max_ of 15.4 nmol P_i_ min^−1^ mg^−1^ protein, the soluble ATPase domain alone, however, appeared largely unaltered compared to the previously assayed 150 mM high‐sodium condition (Table 1). Likewise, variation of the Ca^2+^ and K^+^ concentrations in the assay showed independence of the PfATP4 activity from these cations (Figure 3C,D). The activity was affected, however, by the buffer pH, exhibiting lower hydrolysis rates in the acidic range and an optimum around pH 7.2 (Figure 3E) as seen before with native PfATP4 derived from parasite membranes (Rosling et al. 2018).

Eventually, we evaluated the effect of five known PfATP4 inhibitors from the malaria box representing different chemotypes (MMV006764, MMV007275, MMV011567, MMV665826 und MMV665949) (Spillman and Kirk 2015) plus vanadate as a broad‐spectrum ATPase inhibitor (Figure 3F). None of the malaria box compounds inhibited ATPase activity, which is in line with the assumption that the MMV compounds bind in the transmembrane domain of PfATP4 as deduced from drug‐selected resistance strains (Spillman and Kirk 2015). However, vanadate led to an inhibition of PfATP4 activity to 60% at a 9‐fold access over ATP.

### 2.3 Structure Prediction of the N and P′/P Domains of PfATP4 With Bound ATP and Mg^2+^

**FIGURE 4:**
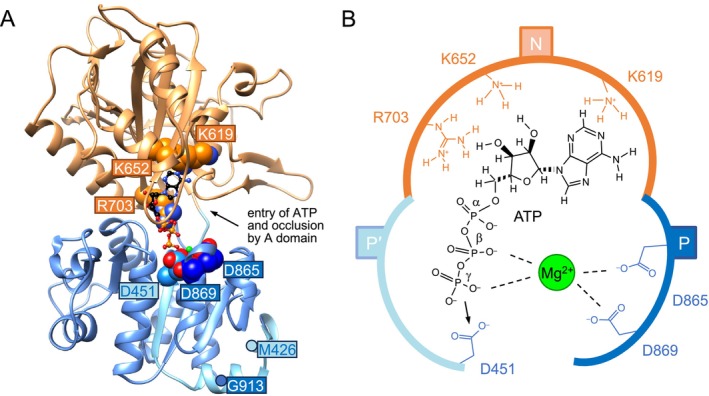
Structure prediction of PfATP4 Met426‐Gly913. (A) AlphaFold 3 model showing the N (orange) and P′/P (light blue/blue) domains with bound ATP (ball and stick), and Mg^2+^ (green). The basic and acidic residues at the ATP binding site are depicted as spheres and labeled. (B) Putative binding mode of ATP and Mg^2+^ in the N and P′/P domains. The phosphate of ATP is supposed to be transferred to Asp451 in native PfATP4 during hydrolysis.

We set out to identify P‐type ATPase‐typical basic and acidic residues in the nucleotide binding (N) and phosphorylation (P′/P) domains of the soluble PfATP4 Met426‐Gly913 construct. Therefore, we employed AlphaFold 3 (Abramson et al. 2024), which allows one to predict ab initio protein folds in the presence of interacting biomolecules and ions. The obtained model (Figure 4A) is conclusive in terms of the mutual orientation of the N and P′/P domains, and a close 10 Å distance of the Met426/Gly913 residues, which is required in the native protein due to their presence at the ends of the neighboring transmembrane helices 4 and 5. Further, the basic Lys619, Lys652, and Arg703 of the N domain, as well as the acidic Asp451, Asp865, and Asp869 of the P′/P domain were placed correctly around the purine/ribose and the triphosphate moieties of ATP; the acidic residues coordinated a Mg^2+^ ion (Figure 4B). Comparison of the N and P domain structures of PfATP4 with the *Saccharomyces cerevisiae* ENA1 type IID P‐type ATPase (Haro et al. 1991) yielded the same general fold (Figure S2).

In the following, we experimentally addressed the role of these sites by point mutations of the respective DNA codons and subsequent protein production, purification, and functional ATPase assays.

### 2.4 Replacement of Conserved Basic Residues in the N Domain

**FIGURE 5:**
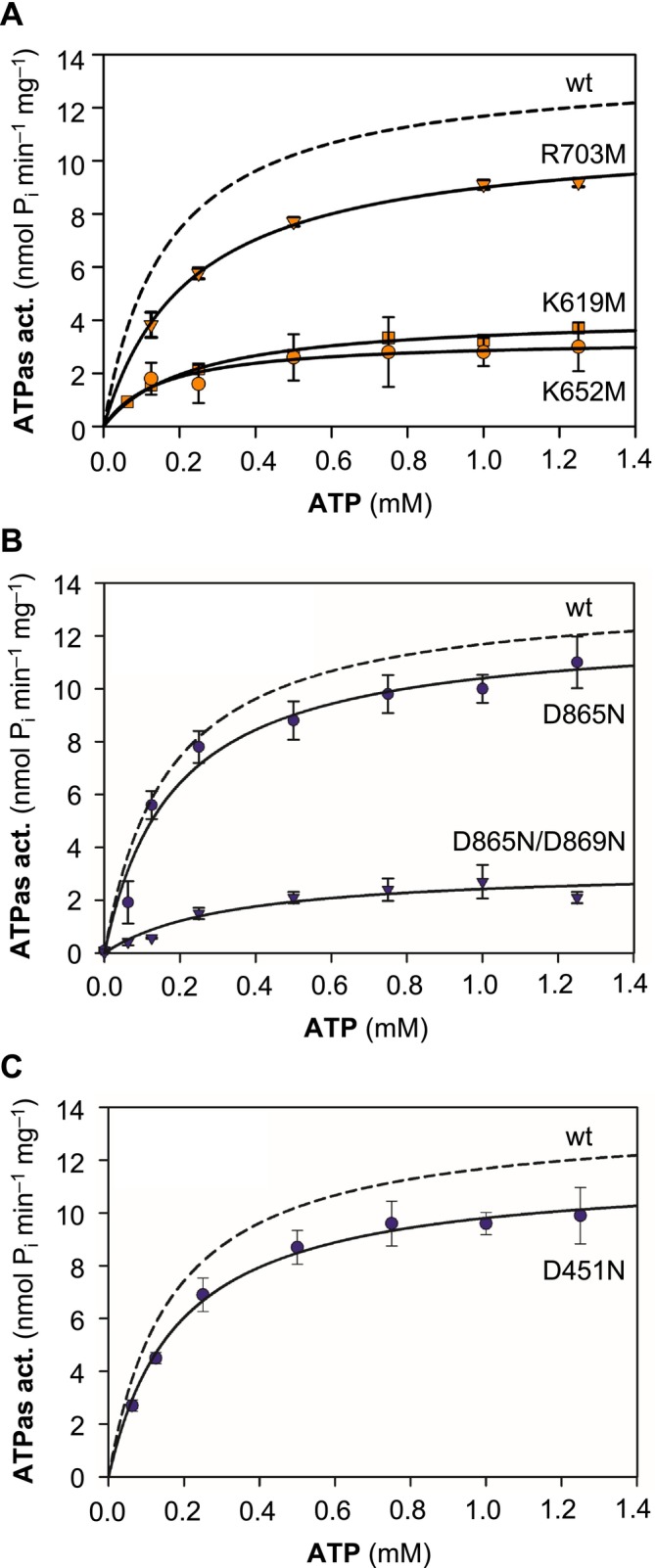
Effect of selected point mutations on the catalytic activity of PfATP4 Met426‐Gly813. (A) ATPase activity after single replacements of basic residues (K619M, K652M, R703M) of the N domain by neutral methionine. (B) ATPase activity with replaced acidic Asp865 and Asp865/Asp869 by neutral asparagine of the P domain. (C) ATPase activity with replacement of the putative γ‐phosphate receiving Asp451 by neutral asparagine in the P′ domain. Activity of the wildtype protein (from Figure 2B) is indicated as a dashed line for comparison. Data show mean ± SEM (*n* = 3–6) except for D865,869 N and D451N (mean ± span; *n* = 2).

First, we modified the basic amino acid residues within the N domain. We replaced conserved basic amino acids in the soluble PfATP4 construct that putatively bind the purine moiety of the ATP substrate (Lys619, Lys 652, Arg703) individually (Figure 5A) and in combination (Figure S3).

Replacement of each of the three basic residues by similarly sized but uncharged methionine decreased the substrate‐dependent rate of ATP hydrolysis by a factor of about 4 for K619M and K652M (Figure 5A). The respective v_max_ values were 4.2 and 3.3 nmol P_i_ min^−1^ mg^−1^ protein (Table 1). The substrate binding affinity decreased only slightly in the lysine single mutants (0.23 and 0.15 mM), whereas the K619,652 M double mutant exhibited a clearly lower affinity of 0.43 mM (Figure S4 and Table 1). The replacement of Arg703 by methionine, in turn, had a lower effect, decreasing the enzyme activity by about one quarter (Figure 5A and Table 1).

### 2.5 Exchange of Acidic Residues of the Mg^2+^ Coordination Site in the P Domain

Replacement of the putative Mg^2+^ coordinating Asp865 and Asp869 in the P domain by the respective neutral amide asparagine (Figure S5) gave a differentiated picture. Eliminating the negative charge of Asp865 yielded a fully functional soluble PfATP4 construct (Figure 5B) with similar K_m_ (0.18 mM) and v_max_ values (v_max_ = 12.3 nmol P_i_ min^−1^ mg^−1^ protein) as the unmodified protein (Table 1). The respective construct with a single Asp869 replacement failed to express in the *E. coli* system and was therefore not available for analysis. However, the protein carrying a joint mutation of Asp865 and Asp869 to asparagine was produced by the cells. The double exchange decreased the ATPase activity to the same low level as seen before with the mutants of the N domain (K_m_ 0.33 mM, v_max_ 3.2 nmol P_i_ min^−1^ mg^−1^ protein; Table 1).

### 2.6 ATP Hydrolysis Occurs Even in the Absence of the γ‐Phosphate Receiving Asp

Eventually, we replaced the putative γ‐phosphate accepting Asp451 of the P′ domain with asparagine. Somewhat unexpectedly, this had no discernible influence on the ATPase activity compared to the unmodified wild‐type construct (Figure 5C). Also, K_m_ (0.18 mM) and v_max_ (11.6 nmol P_i_ min^−1^ mg^−1^ protein) appeared unaffected (Table 1). These data suggest that the inorganic γ‐phosphate moiety can be released directly into the surrounding buffer without previous binding to Asp451. Consistent with the results obtained before, the joint replacement of Asp451 with the Mg^2+^ coordination sites Asp865 and Asp869 (Figures S5 and S6) resulted in decreased ATPase activity (K_m_ 0.14 mM, v_max_ 3.9 nmol P_i_ min^−1^ mg^−1^ protein; Table 1).

## 3 Discussion

In this study, we were able to heterologously produce the ATPase domain of PfATP4 as a soluble protein using bacterial expression and investigated the biochemical and kinetic properties in vitro.

Our findings indicate that the cytosolic domain exhibits ATPase activity in the same order of magnitude as the native PfATP4, as described in previous studies with plasmodial membrane preparations (Rosling et al. 2018). Although this comparison is hampered by the fact that, in the membrane preparations holding the native protein, activity is referred to the total protein content rather than the isolated ATPase, as in our case. With the same caveat, the obtained K_m_ value was similar to those determined in the parasite, showing that the substrate affinity was unchanged, which is indicative of a conserved native protein fold. Notably, ATP hydrolysis was strongly Mg^2+^ dependent, which is common to P‐type ATPases (Apell et al. 2017; Palmgren and Nissen 2011; Kühlbrandt 2004; Dyla et al. 2020). The curve shape of the P_i_ release rate, that is, culmination at a 2.5 mM Mg^2+^ followed by a flat linear decline at higher concentrations, is in line with a previous study examining the Mg^2+^ dependence of the rabbit Na^+^, K^+^ ‐ATPase (Apell et al. 2017).

Unlike native PfATP4, the isolated catalytic domain was found to be unaffected by altered sodium levels. A potential explanation might be in the detachment of the soluble protein from the transmembrane domains. Under physiological conditions, PfATP4 is exposed to a steep transmembrane sodium gradient and subjected to conformational changes in the membrane‐spanning domains induced by ion binding during the transport cycle. The soluble N/P domain construct is decoupled from these events, possibly rendering it independent from Na^+^ ions.

We further showed that the hydrolysis activity of the cytosolic domain is selective for ATP, thereby excluding the possibility of unspecific dephosphorylation of other nucleotides. Like the maintained binding affinity, the ATP substrate selectivity indicates that the genuine folding and function of the nucleotide binding and phosphorylation domains are preserved in vitro and amenable to studies apart from the transmembrane domain.

The tested PfATP4 inhibitors showed no activity on the isolated ATPase domain. The inhibitor binding sites are thought to reside in the transmembrane domain of PfATP4 because mainly here resistance mutations were selectable in parasite cultures. Even though two of the identified mutations, that is, P437S and E895K, fall within the boundaries of the soluble PfATP4 expression construct, the actual ATP binding site remained free of mutations (Spillman and Kirk 2015). Setting up a drug screening assay with the isolated soluble ATPase domain of PfATP4 would certainly be straightforward and well feasibl; however, it seems unlikely that suitable inhibitors with the required specificity for drug development would be found due to the highly conserved layout of the ATP binding site.

In order to specifically disturb the catalytic mechanism, we mutated conserved residues in the N and P′/P domains that have been reported to be crucial for substrate binding and dephosphorylation (Jorgensen et al. 2003; Kubala et al. 2003). Single site mutations of Lys619 and Lys652 caused a strong decrease in ATPase activity, providing evidence for their essential role in substrate binding. Moreover, we can conclude that dephosphorylation in the P domain is preceded by site‐specific binding of ATP within the N domain. Since joint replacement did not further reduce the ATPase activity, all three basic residues contribute essentially to ATP binding. However, further amino acid residues take part in substrate binding and selection.

Joint mutation of the two putative Mg^2+^ coordination sites Asp865 and Asp869 resulted in a substantial reduction of the ATPase activity, whereas the enzyme maintained its activity when only Asp865 was mutated. This confirms the involvement of these aspartate residues in Mg^2+^‐dependent ATP hydrolysis, most likely by properly orienting the triphosphate moiety within the P domain.

Mutation of the Asp451 maintained the ATPase functionality of PfATP4 without the possibility of transferring the γ‐phosphate to the P′ domain. A previous study on the cytosolic ATPase catalytic domain of the mouse Na^+^/K^+^‐ATPase (Krumscheid et al. 2004) equally showed that the release of the γ‐phosphate is possible without the formation of a P′ phosphointermediate. In native full‐length P‐type ATPases, dephosphorylation of the P′ phospho‐aspartate is conveyed by a conserved glutamate residue in the actuator domain connecting ATP hydrolysis to the transmembrane transport process (Olesen et al. 2004). These phospho‐transfers occur within the protein environment and are shielded from the surrounding bulk water. Since the occluding A domain is absent in our construct, the P′/P domain is accessible to water, most likely allowing for hydrolysis even in the absence of an aspartate.

Taken together, the obtained kinetic and selectivity properties of the cytosolic domain of PfATP4 are consistent with results from studies of native PfATP4 and other P‐type ATPases. The cell‐free analysis of the pure soluble PfATP4 catalytic domain presented in this study is a straightforward approach that will enable biochemical characterization under defined and adjustable experimental conditions.

## 4 Methods

### 4.1 Expression Construct and Mutagenesis

A DNA construct encoding the cytosolic loop of PfATP4 (UniProt #A0A143ZZK9) from Met426 to Gly913 connecting the predicted transmembrane spans 4 and 5 was commercially obtained (Genscript) in a codon‐optimized form for *E. coli* expression (Figure S1). The open reading frame was ligated into the pET21a vector using Spe I and Xho I (Novagene). Primers used for site‐directed mutagenesis are shown in Table S1.

### 4.2 Expression in *E. coli* and Cell Disruption

*E. coli* harboring the expression plasmid were incubated at 37°C at 220 rpm shaking until an OD_600_ of 0.6–0.8 was reached. Expression was induced with 1 mM IPTG, and the cells were further grown at 18°C and 220 rpm for 16–20 h. Cells were harvested by centrifugation at 6500 *g*, 4°C for 15 min, washed, and resuspended with 50 mM Tris buffer, pH 7.4, supplemented with 10% glycerol and protease inhibitor (complete, Roche Diagnostics) using 15 mL per 5 g cell pellet. Cell disruption was carried out using the French Press cell disrupter (FA‐078, Thermo Fisher Scientific) at 20.000 psi, 4°C, 3 passages. The lysate was subjected to centrifugation at 30.000 *g*, 4°C for 30 min, and the supernatant was used for subsequent affinity chromatography.

### 4.3 Immobilized Metal Ion Chromatography

The supernatant from disrupted *E. coli* cells was filtrated (Ø 0.45 μm) and incubated with 200 μL of 50% Ni^2+^‐NTA agarose bead slurry (Qiagen) rotating for 2 h at 4°C and 20 rpm. Protein elution was conducted with increasing imidazole concentrations (20–500 mM) in Tris buffer 50 mM, pH 7.2, with 500 mM NaCl using chromatography columns (poly‐prep, Bio‐Rad). Imidazole fractions with the highest content of the protein of interest were pooled and concentrated (Amicon Ultra‐4 with 30 kDa cut‐off; Merck). Protein concentration was determined (Bio‐Rad protein assay; BioRad). Protein fractions were analyzed using SDS‐PAGE and Western Blot. Protein bands were stained with Coomassie Brilliant Blue. For Western blotting, proteins were transferred to PVDF membranes (Hybond, GE Healthcare) and detected using an anti‐penta‐His mouse antibody (Qiagen) and secondary goat‐anti‐mouse antibodies (Jackson ImmunoResearch), each diluted 1:5000 for detection (Clarity Western ECL Substrate; Biorad) in a ChemoStar Touch cabinet (Intas).

### 4.4 ATPase Assays

ATPase activity was quantified in 96‐well format by measuring the release of P_i_ from ATP hydrolysis at 37°C over 30 min. The reaction mixtures consisted of 10 μg of purified protein in 50 μL 50 mM Tris, pH 7.2, 150 mM NaCl, plus 2 mM MgCl_2_ (if not indicated otherwise) and increasing concentrations (0–1.4 mM) ATP. Reaction mixtures were supplemented with different cations and inhibitors as indicated. The reactions were terminated by adding 100 μL of assay reagent (Biomol green phosphate detection kit; Enzo Life Sciences) to each well for 30 min. Absorbance at 620 nm was measured using a microplate reader (Infinite F200; Tecan). The background signal was determined from wells containing all reaction components except protein and was subtracted.

### 4.5 Structure Prediction and Display

The protein fold of PfATP4 Met426‐Gly913 was predicted with AlphaFold 3 (Abramson et al. 2024) using the online AlphaFold Server. For predicting interactions with the substrate, ATP was set as a ligand and Mg^2+^ was set as an ion entity in the process. The protein structure was displayed using Chimera (Version 1.16; Pettersen et al. 2004).

## Author Contributions

**Timo Beyer:** methodology, investigation, validation, visualization, writing – original draft. **Jesko Caliebe:** methodology, investigation, validation. **Lara Kähler:** investigation, validation. **Eric Beitz:** conceptualization, formal analysis, visualization, writing – review and editing, project administration, supervision, resources.

## Conflicts of Interest

The authors declare no conflicts of interest.

## Supporting information


Data S1.

## Data Availability

The data that supports the findings of this study are available in the Supporting Information of this article.
