# Supplementary material for: A Soluble Expression Construct of the Isolated Catalytic Domain of Plasmodium falciparum ATP4 Exhibits ATPase Activity Independent of a γ‐Phosphate Receiving Aspartate
Source: Mol Microbiol. 2025 Mar 17;123(6):479–86. doi: 10.1111/mmi.15358 (PMC12152296; doi:10.1111/mmi.15358)
Supplement: Supplementary file 1 — Data S1. [file MMI-123-479-s001.pdf]

A soluble expression construct of the catalytic domain of *Plasmodium falciparum* ATP4 exhibits ATPase activity independent of the conserved γ-phosphate receiving aspartate

Timo Beyer, Jesko Caliebe, Lara Kähler, Eric Beitz

Figure S1 Optimized coding sequence of the PfATP4 Met427-Gly913 expression construct

Figure S2 AlphaFold 3 structure comparison of PfATP4 with *S. cerevisiae* ENA1

Figure S3 Expression and purification of soluble PfATP4 N domain mutants

Figure S4 ATPase activity of soluble PfATP4 N domain double mutant

Figure S5 Expression and purification of soluble PfATP4 P'/P domain mutants

Figure S6 ATPase activity of the soluble PfATP4 P'/P domain triple mutant D451N/D865,869N

Table S1 Mutation primers for PfATP4 Met427-Gly913 variants

actagtATGGTTAAGAAAAACGCGAACGTTCGTAAACTGCCGGCGGTGGAGACACTTGGTTG
CTGTAGCGTTATCTGTTCTGATAAAACCGGCACGCTGACTGAAGGTAAGATGACCGCCATCA
ATGCTGTGACCATTTGCAAAAACTCCTCGCTGAGCGACGAAAATAACAAGTTAACGAAAACG
TTCGATTTCTACCCAACCAAAGGGTTCGAGCCGTGTGGTGGTCTGTTTGATTCGAATGAACT
GACCTCAGAGAAGAAGAAGGAGATTGTGATCGCAAAAAATCAGAACACGAGCTACGATAAAG
TTCTGTACAACTACGGTAATCCGAGCAACAAGTCCGTTATCGTTGATAAGACACGTTCCCTG
ATGTTTGCGGCGTATCTGAATTCTTACGACACCACCCTGAGCCGCGATCCGAAGACTCTGAA
GTGGGGAATTCACGGCAACATGAGCGAAGGACCGATTGTAGTCGCAGCGGCTAAGGTTGGCT
ATTCTTTCATCAACAACCCGAATCATAAAAGCTACTTGGATAATTTCCAGCGTCTGGACGAT
CTGGAAGTGACGTTCAACTCTAGCCGCAAAATGAAGATCACCTTCTATAAACTGAAGACGGT
AAATGTCTTCGAAAACGTGTACCTGGACAAACCGGGTAAGGTTTACACCCACGTTGCATTAA
TTAAAGGTGCACCGGATCGTCTGCTCGACCGTAGCACCCATCTGCTGGAAGAAACCAGCATG
AAAAAAGTGCAGGTGTCCTGGAATTCCACCATCACCCAAGAGGAGCGCAACGTCCTCATCAA
GAAAAACCTGGAGCTGTCCCAGAAAGCGTTGCGCGTGTTGAGCATCTGCATCAAACCGTTGA
CTGACCAAAACATCGAGGAGCTGAAAAAGCTGGAGGACGCAGACGAACGTCTGAAGTATGTG
AATTATGACGAAAACGGCGGTTTTATCCCTATGGGTTACGTTGCTTCTTTCGACCCGCCGCG
TCCAGGGGTTAAGGAGGCGATTCAGACCTGCCGTGAAGCTCAGGTGAAAGTTATTATGATTA
CCGGTGATCAAAAACCGACCGCCGTGGCGATCGGTAAATTAATCGGCCTTATCGAGGAAAAA
AGCGAGCAGGTTGAAGACATCAATAGTCTGGCGATTGAGTGCAGCGAACTTCACATTAACAA
GAACCCGAACGAGCCGATTTTACCGAATGACCAACTGGACGAGTTTACCGATAAAATCCTGA
TATACAGTCGTGCGCAACCGGAAGACAAAATCACGATCGTTCAGAGCCTGAAGCGCAAAGGC
TACTTGGTTGCTATGACTGGTGACGGTGTGAATGACGCACCGGCCCTGAAGGCGGCGGACAT
CGGCGTCGCCATGGGTATTAATGGTACCGAAGTTGCGAAAGGCGCTAGCGAGATGATTTTGA
TCGATGATAACTTCTGCACCGTTGTGAGCGCGATCGACGTGGGCctcgag

Figure S1. Codon-optimized DNA sequence encoding PfATP Met427-Gly913. The added 5' and 3' cloning sites Spe I and Xho I are in lower case and underlined.

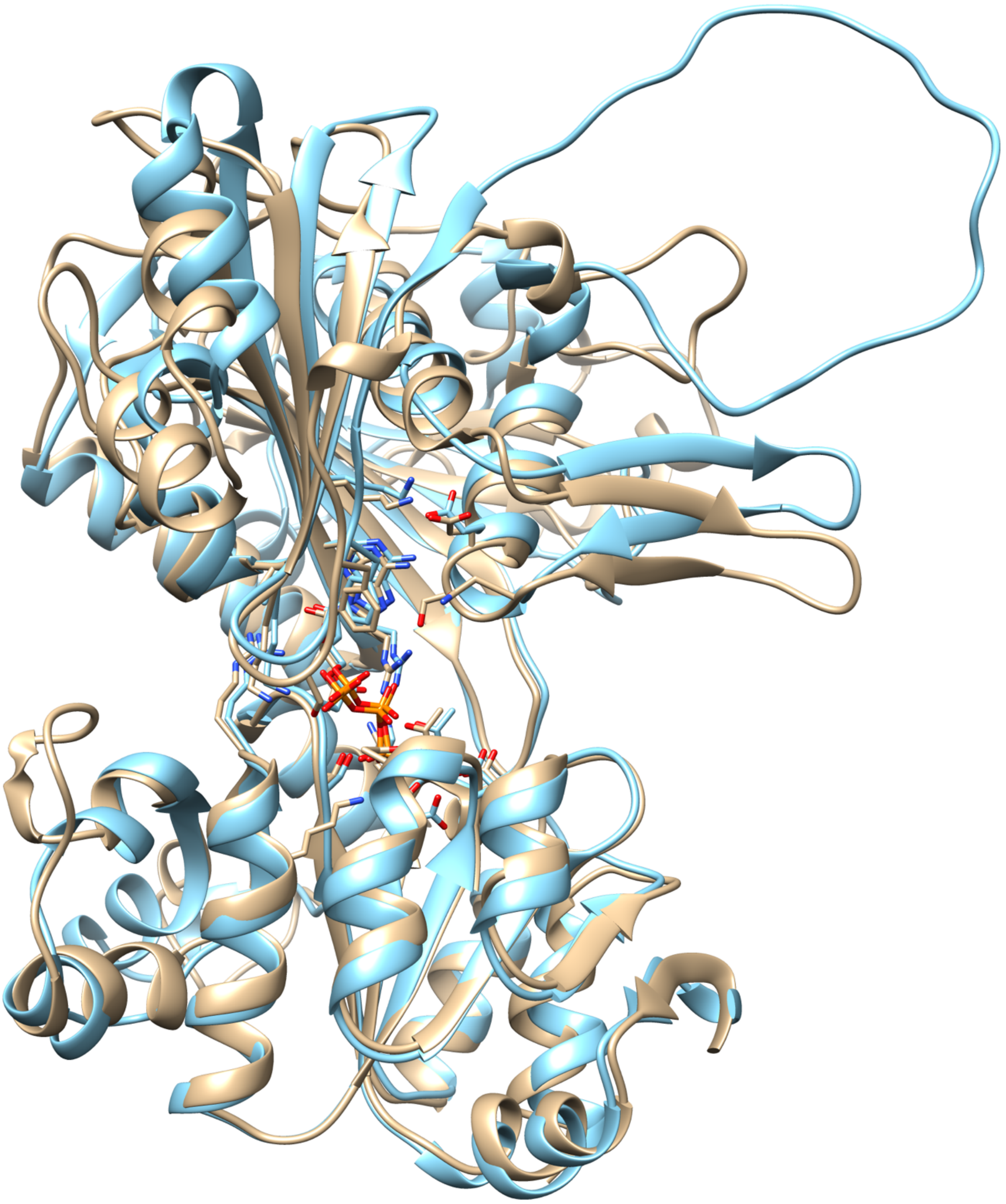

Figure S2. AlphaFold 3 structure comparison of the N and P domains of PfATP4 (beige) and *Saccharomyces cerevisiae* ENA1 (blue; UniProt# P13587). The bound ATP and interacting residues are shown as sticks.

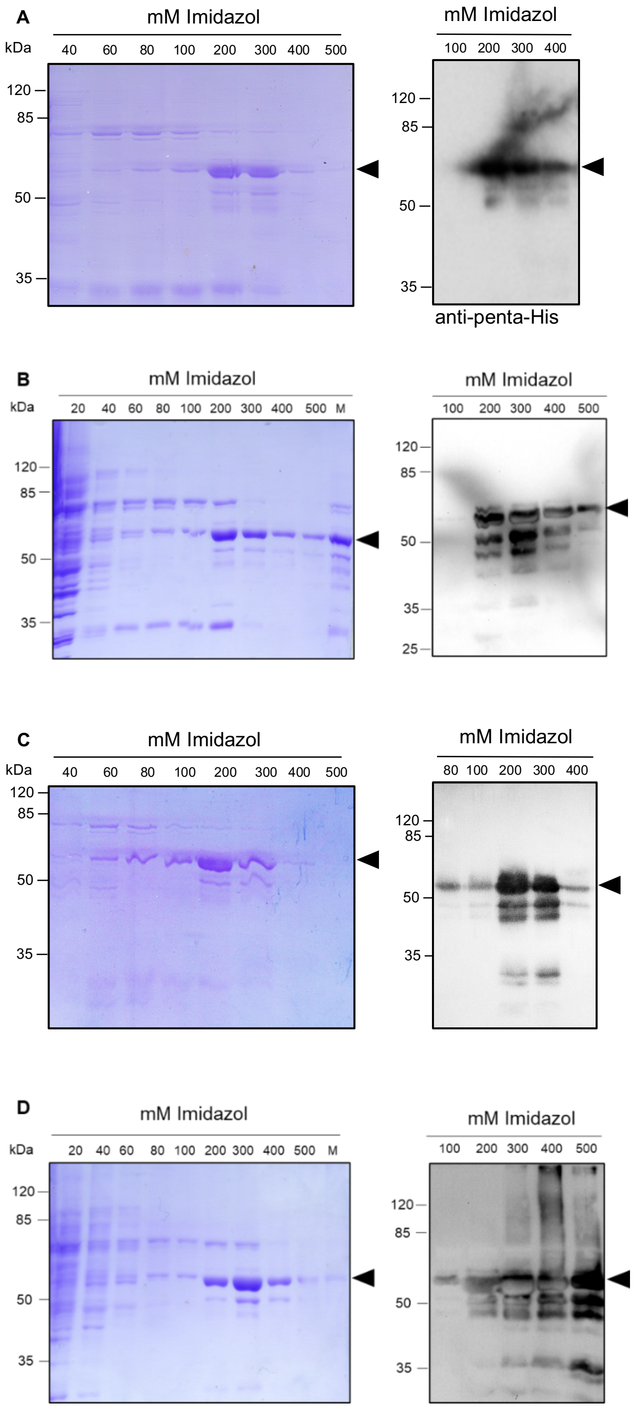

Figure S3. Expression and purification of soluble PfATP4 N domain mutants. Shown are the Coomassie-stained elution profiles and the respective Western blots of K619M (A), K652M (B), R703M (C), and K619,652M (D). Arrow heads indicate the protein of interest at the expected size of 54 kDa.

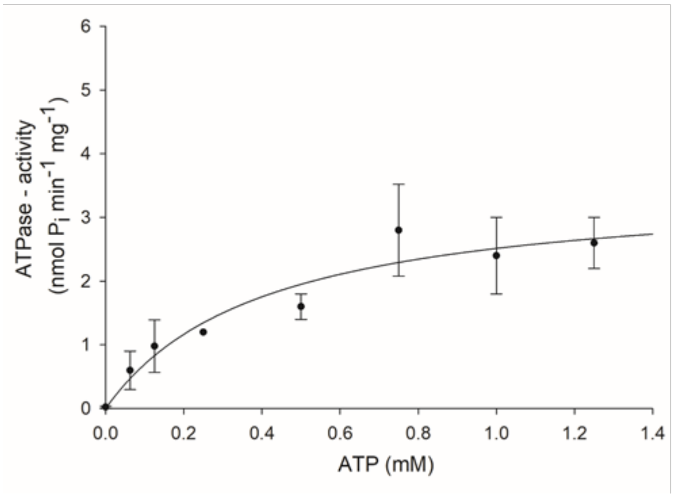

Figure S4. ATPase activity of soluble PfATP4 N domain double mutant. Hydrolysis activity was measured for K619,652M. Data are shown as mean ± SEM, n = 3. Values for K_m and v_{max} determined with the respective Michaelis-Menten curve fit are shown in Table 1 of the main paper.

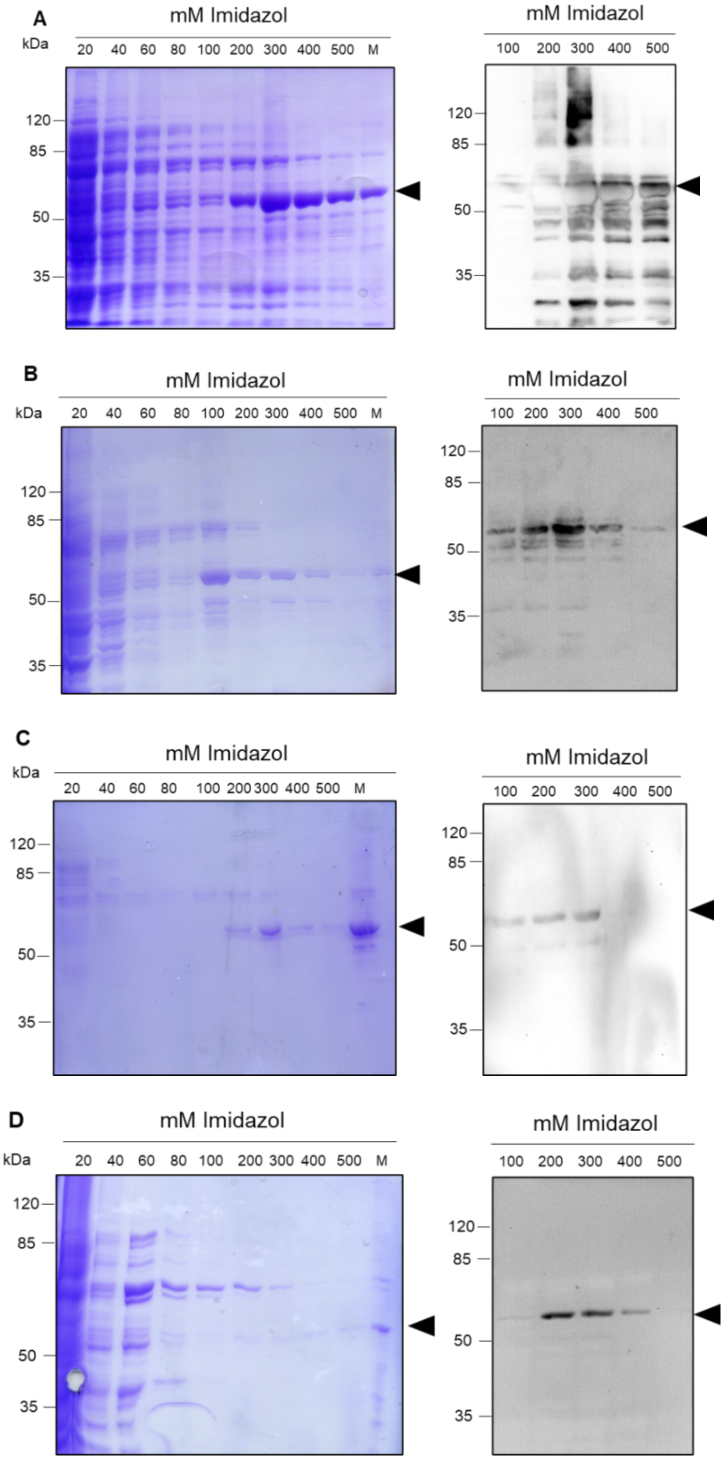

Figure S5. Expression and purification of soluble PfATP4 P'/P domain mutants. Shown are the Coomassie-stained elution profiles and the respective Western blots of D451N (A), D865N (B), D865,869N (C), D451N/D865,869N (D). Arrow heads indicate the protein of interest at 54 kDa.

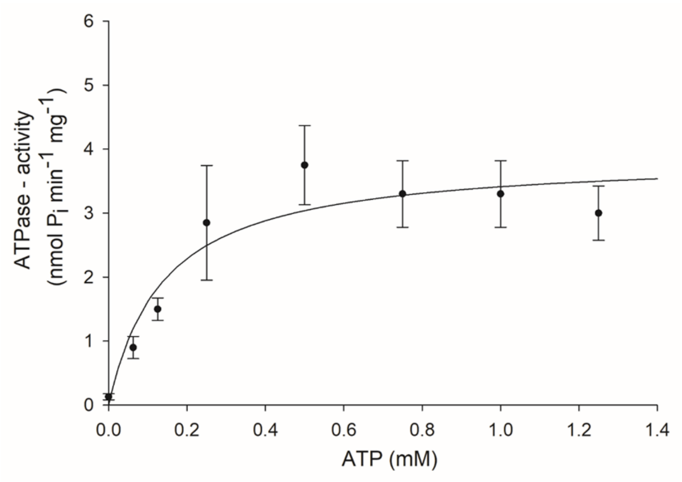

Figure S6. ATPase activity of the soluble PfATP4 P'/P domain triple mutant D451N/D865,869N. Data are shown as mean ± SEM, n = 4. Values for K_m and v_{max} determined with the respective Michaelis-Menten curve fit are shown in Table 1 of the main paper.

Table S1. Mutation primers for PfATP4 Met427-Gly913 variants. Noncomplementary nucleotides are underlined.

D865N s	5′ GTT GCT ATG ACT GGT AAC GGT GTG AAT GAC GCA 3′
D865N as	5′ ACC CGC AAA GGC TAC TTG GTT GCT ATG ACT 3′
D865,869N s	5′ GGT AAC GGT GTG AAT AAC GCA CCG GCC CTG AAG 3′
D865,869N as	5′ ATT CAC ACC GTT ACC AGT CAT AGC AAC CAA 3′
K619M s	5′ TTC AAC TCT AGC CGC ATG ATG AAG ATC ACC TTC 3′
K619M as	5′ GCG GCT AGA GTT GAA CGT CAC TTC CAG ATC 3′
K652M s	5′ CAC GTT GCA TTA ATT ATG GGT GCA CCG GAT CGT 3′
K652M as	5′ AAT TAA TGC AAC GTG GGT GTA AAC CTT ACC 3′
R703M s	5′ TCC CAG AAA GCG TTG ATG GTG TTG AGC ATC TGC 3′
R703M as	5′ CAA CGC TTT CTG GGA CAG CTC CAG GTT TTT 3′
D451N s	5′ AGC GTT ATC TGT TCT AAT AAA ACC GGC ACG C 3′
D451N as	5′ G CGT GCC GGT TTT ATT AGA ACA GAT AAC GCT 3′
